# Whole-body [^18^F]-FDG-PET/MRI for staging of pediatric non-Hodgkin lymphoma: first results from a single-center evaluation

**DOI:** 10.1186/s13550-021-00804-8

**Published:** 2021-07-03

**Authors:** L. Kurch, R. Kluge, O. Sabri, L. Fischer, S. Wendt, H. Graf Einsiedel, S. Starke, J.-S. Kühl, H. Christiansen, F. W. Hirsch, I. Sorge, C. Roth

**Affiliations:** 1 Department of Nuclear Medicine, University Hospital Leipzig, Leipzig, Germany; 2 Department of Pediatric Oncology, Hematology and Hemostaseology, University Hospital Leipzig, Leipzig, Germany; 3 Institute of Pediatric Radiology, University Hospital Leipzig, Leipzig, Germany

**Keywords:** [^18^F]-FDG-PET/MRI, Hybrid imaging, Pediatric non-Hodgkin lymphoma, Pediatric oncology

## Abstract

**Aim:**

In 2015, the revised International Pediatric Non-Hodgkin Lymphoma Staging System was published. It mentions [^18^F]-FDG-PET/MRI as the latest method to perform whole-body imaging. However, supporting data are pending. Our aim was to investigate the performance of whole-body [^18^F]-FDG-PET/MRI in pediatric non-Hodgkin lymphoma patients by using a limited number of MRI sequences.

**Materials and methods:**

Ten pediatric patients with histologically proven non-Hodgkin lymphoma underwent whole-body [^18^F]-FDG-PET/MRI at staging. The retrospective analysis included three steps: First, [^18^F]-FDG-PET and MR scans were evaluated separately by a nuclear medicine physician and a pediatric radiologist. Nineteen nodal and two extranodal regions as well as six organs were checked for involvement. Second, discrepant findings were reviewed together in order to reach consensus. Third, [^18^F]-FDG-PET/MRI findings were correlated with the results of other clinical investigations.

**Results:**

Of the 190 lymph node regions evaluated, four were rated controversial. Consensus was reached by considering metabolic, functional and morphologic information combined. Concordantly, [^18^F]-FDG-PET and MRI detected Waldeyer’s ring involvement in two patients whose Waldeyer’s ring was negative on clinical assessment. In four patients MRI showed pleural effusion. However, in only two of them an increased glucose metabolism as a reliable sign of pleural involvement was detectable. In six patients [^18^F]-FDG-PET and MRI detected skeletal lesions although bone marrow biopsy was positive in only one of them.

**Conclusion:**

Despite the small number of cases evaluated, whole-body [^18^F]-FDG-PET turned out to be a valuable tool for staging of pediatric non-Hodgkin lymphoma.

## Introduction

Lymphoma is the third most common malignancy in children and adolescents. About 60% account for non-Hodgkin lymphoma (NHL) and 40% for Hodgkin lymphoma (HL). In the past 40 years, major advances were achieved in the fields of cytotoxic drug development, radiation oncology, pathology and imaging technologies. Each of them contributed to a more individualized treatment.

The role of the latest whole-body imaging technologies, [^18^F]-FDG-PET/CT and [^18^F]-FDG-PET/MRI, has been studied, but much more extensively in pediatric HL than in pediatric NHL [1]. Yet, according to the PubMed database, to date there is no publication available which exclusively addresses the application of simultaneous [^18^F]-FDG-PET/MRI in pediatric NHL (last PubMed query: April, 14, 2021).

In 2015, the revised International Pediatric non-Hodgkin Lymphoma Staging System (IPNHLSS) was published and since then replaces the St. Jude classification by Murphy from the 1980s [2]. The main publication on the revised IPNHLSS also considers advances in imaging technologies and points out the efficiency and potency of hybrid imaging such as [^18^F]-FDG-PET/CT and [^18^F]-FDG-PET/MRI [2]. [^18^F]-FDG-PET/MRI seems to be especially promising since it can depict metabolic (i.e., the degree of glucose turnover), functional (i.e., the diffusion movement of water molecules) and morphological properties of tumor lesions at the same time. Considering all three aspects combined could be substantial for accurate characterization of lesions and may reduce the deficiencies of each modality when used separately. Furthermore, radiation exposure can be saved when replacing CT imaging by MRI.

However, whole-body imaging with [^18^F]-FDG-PET/MRI represents a compromise between the acquisition of all potentially possible MR sequences and a reasonable overall scan duration [3]. The overall scan duration is the determining factor with regard to compliance of young patients. It should not exceed 60 min [3].

The aim of this retrospective single-center evaluation was to investigate the performance of whole-body [^18^F]-FDG-PET/MRI in pediatric NHL patients by using a limited number of MRI sequences.

## Patients, materials and methods

### Patients

Between April 2012 and November 2019 ten pediatric NHL patients received whole-body [^18^F]-FDG-PET/MRI for initial staging.

Written informed consent was obtained from all patients and/or their legal guardians for scientific evaluation of imaging data before inclusion into this retrospective evaluation.

The data evaluation was approved by the local Ethics Committee.

### Patient data and investigations beyond [^18^F]-FDG-PET/MRI

For each patient, age, sex, histology, the presence of B symptoms and the level of lactate dehydrogenase (LDH) were documented. Wherever available, the results of ear, nose and throat (ENT) examination, lumbar puncture, bone marrow (BM) biopsy, thoracentesis as well as computed tomography (CT) of the chest and ultrasound of the abdomen were considered for correlation with the [^18^F]-FDG-PET/MRI images.

### [^18^F]-FDG-PET/MRI imaging

Images were acquired on a 3 Tesla Biograph mMR (Siemens, Erlangen, Germany) which had been installed in 2011.

Prior to the [^18^F]-FDG injection all patients fasted for at least 6 h.

To avoid activation of brown adipose tissue all patients were warmed for at least 30 min before [^18^F]-FDG injection. In addition, the unselective beta-blocker propranolol (1 mg/kg, maximum dose = 40 mg) was administered approximately one hour before tracer injection.

The upper limit of [^18^F]-FDG activity to be administered was determined by the EANM dosage calculator [4]. Dosing reduction ranged from 24 to 67% of the recommended upper activity limit in nine patients (Table 1). In one patient with marginal overweight the administered activity reached the recommended upper limit (Table 1).

**Table 1:** Patient characteristics [^18^F]-FDG-PET/MRI parameters and results of further staging examinations

Pat	Age	Histology	ENT_clin_	TC	LP	BMB	US_abdomen_	^18^F-FDG_adm_	^18^F-FDG_recom_	Δ_inject/scan_	Sedation	Images performed	Result of PET/MR scan	Figure
1	15 years	Burkitt	n.d	n.d	−	−	Splenomegaly, otherwise US -	212 MBq (− 39%)	348 MBq	90 min	No	WB PET; WB MR T2-TIRM_cor + trans_; WB DWI_trans_	Nodular lymphoma involvement on both sides of the diaphragm	
2	16 years	DLBCL_oss_	−	−	−	+	−	276 MBq (− 24%)	363 MBq	90 min	No	WB PET; WB MR T2-TIRM_cor + trans_; WB DWI_trans_	Nodular lymphoma involvement on both sides of the diaphragm, extensive involvement of the left pelvic bones with cortical bone destruction and infiltration of adjacent muscles	Figure 8i–l
3	6 years	DLBCL	−	n.d	−	−	−	64 MBq (− 49%)	126 MBq	135 min	Yes	WB PET; WB MR T2-TIRM_cor + trans_; WB DWI_trans_	Nodular lymphoma involvement on both sides of the diaphragm	Figures 1, 3
4	7 years	DLBCL	−	n.d	−	−	−	91 MBq (− 39%)	148 MBq	110 min	No	WB PET; WB MR T2-TIRM_cor + trans_; WB DWI_trans_	Nodular lymphoma involvement on both sides of the diaphragm	
5	10 years	DLBCL_oss_	−	n.d	−	−	−	126 MBq (− 45%)	229 MBq	180 min	No	WB PET; WB MR T2-TIRM_cor + trans_; WB DWI_trans_	Long segmental lymphoma involvement of the left femur with cortical bone destruction and infiltration of adjacent muscles	
6	10 years	T-cell lymphoma	−	n.d	−	−	Bilateral renal involvement, otherwise US -	278 MBq (+ 0%)	277 MBq	110 min	No	WB PET; WB MR T2-TIRM_cor + trans_; WB DWI_trans_	Nodular lymphoma involvement on both sides of the diaphragm including Waldeyer’s ring. In addition bilateral kidney and skeletal involvement (right femur)	Figures 6, 7
7	14 years	Burkitt	−	n.d	−	−	−	162 MBq (− 45%)	292 MBq	45 min	No	WB PET; WB MR T2-TIRM_cor + trans_; WB DWI_trans_	Nodular lymphoma involvement above diaphragm including Waldeyer’s ring. Moreover, focal skeletal involvement	Figures 4, 7, 8a–h
8	15 years	T-cell lymphoma	+	n.d	−	−	Bilateral renal involvement, otherwise US -	67 MBq (− 67%)	200 MBq	70 min	No	WB PET; WB MR T2-TIRM_cor + trans_; WB DWI_trans_	Nodular lymphoma involvement above diaphragm including left pleura, extended bilateral and focal skeletal involvement (left femur). Waldeyer’s ring involvement most unlikely	Figure 2
9	4 years	T-cell lymphoma	+	+	−	−	−	45 MBq (− 51%)	92 MBq	70 min	No	WB PET; WB MR T2-TIRM_cor + trans_; WB DWI_trans_ n.e	Nodular lymphoma involvement above diaphragm and left-sided pleural involvement. Waldyer’s ring involvement most unlikely	Figure 5
10	17 years	DLBCL	−	n.d	−	−	−	239 MBq (− 31%)	348 MBq	80 min	No	WB PET; WB MR T2-TIRM_cor + trans_ WB DWI_trans_ n. d	Nodular lymphoma involvement above diaphragm	

BMB, Result of Bone Marrow Biopsy; Cor, coronal; DLBCL_oss_, Primary Osseous Large B-cell Lymphoma; ENT_clin_, Result of clinical ENT investigation; ^18^F-FDG_adm,_ Administered ^18^F-FDG activity; LP, Result of Lumbar Puncture; min, minutes; n.e., not evaluable; TC, Thoracentesis; Trans, transversal; US_abdomen_, Result of abdominal ultrasound; y, years; Δ_inject/scan_, Time interval from tracer injection to scan start; + , positive for lymphoma; −, negative for lymphoma; Burkitt, Burkitt Lymphoma; DLBCL, Diffuse Large B-cell Lymphoma; DWI, Diffusion Weighted Imaging; f, female; ^18^F-FDG_recom,_ Recommended ^18^F-FDG activity; m, male; n.d., not done; Pat, Patient number; T-cell LL, T-cell Lymphoma; T2-TIRM, T2 weighted Turbo-Inversion Recovery Magnitude; WB, wholebody

Time interval between radiotracer injection and start of the scan ranged from 45 to 135 min for nine patients (Table 1). The variation in injection to imaging time is mainly due to unexpected demand on the scanner throughout daily routine. In one patient the interval was 180 min due to temporary scanner dysfunction (Table 1).

One of the ten patients needed sedation for [^18^F]-FDG-PET/MRI acquisition (Table 1).

[^18^F]-FDG attenuation correction was carried out per section with a MR Dixon sequence as described by Hirsch et al. [3].

Water-sensitive fast inversion recovery sequences (T2-TIRM) with 5 mm in coronal and 4.5 mm in transversal plane were acquired to provide anatomical coverage of the entire body. This was limited to a maximum length of 160 cm (seven stacks with overlap of 30 cm length each). Taller children and adolescents underwent a second scan covering the legs after repositioning.

Respiratory triggering with a belt system was used for image acquisition of the thoracic and upper abdominal region in order to obtain improved image quality and to minimize breathing artifacts.

To reduce artifacts through excessive peristalsis all patients received butylscopolamine approximately 10–15 min before acquisition of the abdominal region was performed (0.3 mg/kg, maximum dose = 20 mg).

Additionally, diffusion-weighted images (DWI) with a slice thickness of 6 mm were acquired if patient compliance was sufficient. This was the case in eight of the ten patients (Table 1). One patient, however, refused an additional DWI whereas a second patient was unable to lie still during DWI acquisition resulting in severe movement artifacts. DWI allowed for calculation of the apparent diffusion coefficient (ADC). ADC cutoff values (B-values) at or less than 800 × 10^–6^ mm^2^/s were regarded as diffusion restriction.

Altogether, the acquisition with its detailed parameters was in accordance with recommendations by Hirsch et al. [3].

### [^18^F]-FDG-PET/MRI data analysis

[^18^F]-FDG-PET/MRI datasets were retrospectively analyzed by an experienced pediatric radiologist (CR) and an experienced nuclear medicine physician (LK), both having more than 10 years’ experience in their respective field. Nineteen lymph node regions, adapted to [5], two extranodal regions [Waldeyer’s ring (WR), pleura] as well as six organs [central nervous system (CNS), lungs, liver, spleen, kidneys and skeleton) were defined for evaluation.

First, [^18^F]-FDG-PET images were evaluated by the nuclear medicine physician and MR images (T2-TIRM coronal and transversal) by the pediatric radiologist. The nuclear medicine physician focused on the aspects of glucose metabolism (intensity and configuration of uptake) whereas the radiologist reviewed aspects of morphology (diameters and intensity of the T2 signals). Thereby, the above-mentioned regions were classified as (a) involved = positive, (b) not involved = negative. The results were documented in separate SPSS tables (SPSS^24^).

In a second step, both physicians reviewed discrepant findings jointly. For this purpose, diffusion weighted MR images were also reviewed whenever available. Consensus was reached by weighing the respective image information ([^18^F]-FDG, T2-TIRM coronal and transversal, diffusion-weighted images) and discussing them with other experienced colleagues.

Third, findings on [^18^F]-FDG-PET/MR images were correlated with the results of other clinical investigations or imaging modalities as mentioned above.

## Results

### Patient characteristics and results of performed investigations beyond whole-body [^18^F]-FDG-PET/MRI

Table 1 shows main patient characteristics and results of staging investigations aside from [^18^F]-FDG-PET/MRI parameters.

Additionally, two of the 10 patients reported B symptoms. Lactate dehydrogenase (LDH), measured upon hospital admission, was highly normal to moderately elevated in most patients. Respective values ranged from 4 to 22 µkat/l (normal values: 1.94–4.78 µkat/l).

 Two of the ten patients underwent chest CT ruling out micro-nodular involvement in both of them.

### [^18^F]-FDG-PET/MRI data analysis

#### Lymph node regions

In 10 patients a total of 190 lymph node regions were evaluated. The results are displayed in Table 2. Altogether, four discrepancies between [^18^F]-FDG and MRI (T2-TIRM transversal, coronal) reading occurred (Table 2) and are described below. Decision making is summarized in Table 3.

**Table 2:** Lymph node regions separated by imaging modality

Lymph node region	Evaluation of MR		Evaluation of PET		Consensus (MR + PET)	
	Negative	Positive	Negative	Positive	Negative	Positive
Right upper neck*	6/10	4/10	5/10	5/10	5/10	5/10
Left upper neck	6/10	4/10	6/10	4/10	6/10	4/10
Right lower neck	6/10	4/10	6/10	4/10	6/10	4/10
Left lower neck	8/10	2/10	8/10	2/10	8/10	2/10
Right clavicular	6/10	4/10	6/10	4/10	6/10	4/10
Left clavicular	7/10	3/10	7/10	3/10	7/10	3/10
Right axillary**	7/10	3/10	8/10	2/10	8/10	2/10
Left axillary**	7/10	3/10	8/10	2/10	8/10	2/10
Right lung hilum	8/10	2/10	8/10	2/10	8/10	2/10
Left lung hilum***	9/10	1/10	8/10	2/10	8/10	2/10
Mediastinum	6/10	4/10	6/10	4/10	6/10	4/10
Splenic hilum	10/10	0/10	10/10	0/10	10/10	0/10
Liver hilum	10/10	0/10	10/10	0/10	10/10	0/10
Mesenterial	8/10	2/10	8/10	2/10	8/10	2/10
Paraaortic	6/10	4/10	6/10	4/10	6/10	4/10
Right iliacal	8/10	2/10	8/10	2/10	8/10	2/10
Left iliacal	7/10	3/10	7/10	3/10	7/10	3/10
Inguinal re	8/10	2/10	8/10	2/10	8/10	2/10
Inguinal li	9/10	1/10	9/10	1/10	9/10	1/10
Sum	142/190	48/190	142/190	48/190	142/190	48/190

* discrepancy during first lymph node evaluation in the right upper neck

** discrepancy during first lymph node evaluation in the right and left axillary

*** discrepancy during first lymph node evaluation left lung hilum

**Table 3:** Summary of the decision-making process in case of discrepancies between ^18^F-FDG-PET and MR concerning lymph node staging

Case (Figure)	1*	2**	3***
^18^F-FDG-PET	++	−	+
MR (TIRM cor/trans)	−	(+)	−
ADC	+	−	−
Final decision	+	−	−

* discrepancy during first lymph node evaluation in the right upper neck

** discrepancy during first lymph node evaluation in the right and left axillary

*** discrepancy during first lymph node evaluation left lung hilum

–not involved, (+) possibly involved, +/++ involved

##### Cervical neck (Fig. 1a–c, Table 3*)

**Fig. 1:**
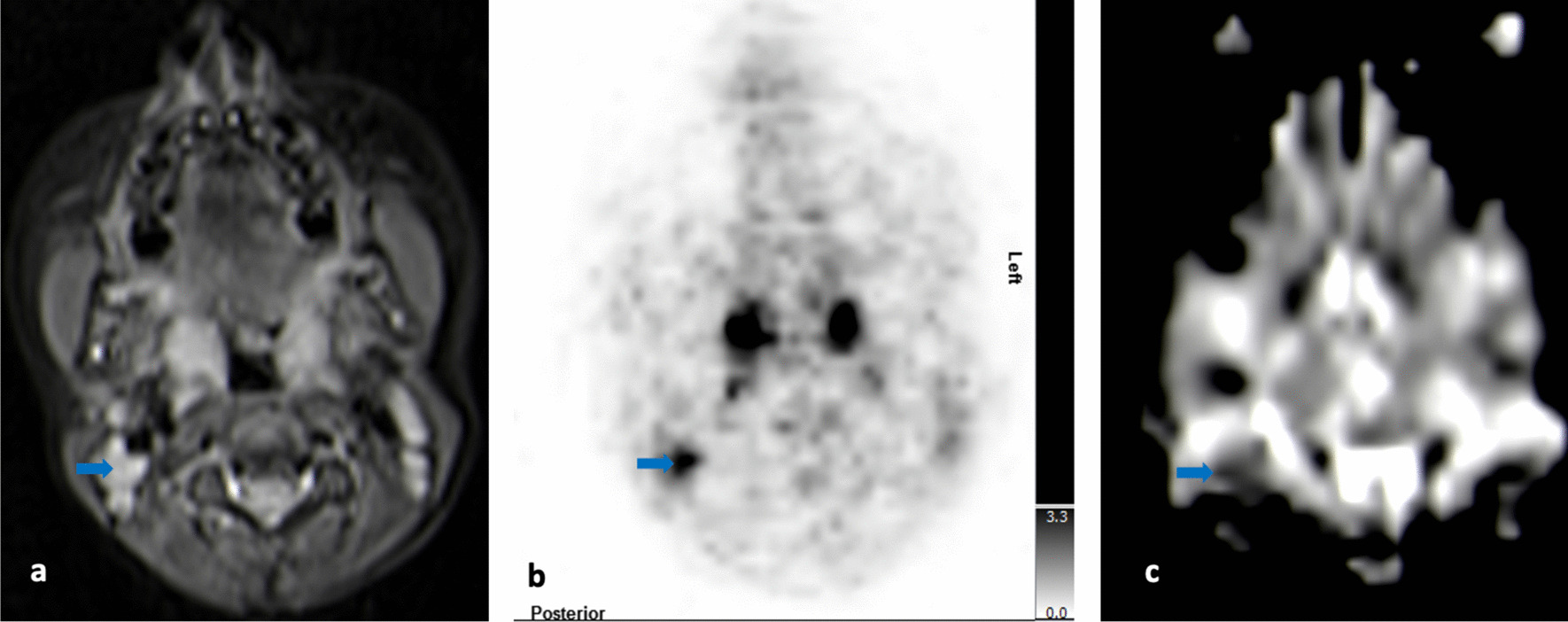
**a**–**c** Upper neck (level II) on T2-TIRM transversal (**a**), [^18^F]-FDG-PET (**b**), and DWI at b-value of 800 (**c**): The blue arrows point to morphologically inconspicuous lymph nodes on the right side (**a**) with increased glucose metabolism (SUV_max_ right: 3.96 versus SUV_max_ left: 1.84) (**b**) and restricted diffusion (ADC right on average: 643 × 10^–6^ mm^2^/s versus ADC left on average: 1480 × 10^–6^ mm^2^/s) (**c**)

In this case, morphologically inconspicuous upper cervical lymph nodes (level II) were detectable on MRI on both sides of the upper neck (lymph node size/right side: 1.8 × 1.6 × 1.2 cm; lymph node size/left side: 1.9 × 1.8 × 0.8 cm) (Fig. 1a), which is not uncommon in children. However, for the lymph nodes on the right side, in contrast to the other side, moderate to markedly increased glucose metabolism was detectable (SUV_max_ right side: 3.96; SUV_max_ left side: 1.84), making these lymph nodes suspicious for lymphoma involvement (Fig. 1b). This was supported by diffusion restriction of the right sided lymph nodes only (ADC right on average: 643 × 10^–6^ mm^2^/s versus ADC left on average: 1480 × 10^–6^ mm^2^/s) (Fig. 1c). In conclusion, the lymph nodes on the right side of the upper neck were determined to be most likely involved.

##### Axillary lymph nodes on the right and left side (Fig. 2a–e, Table 3**)

**Fig. 2:**
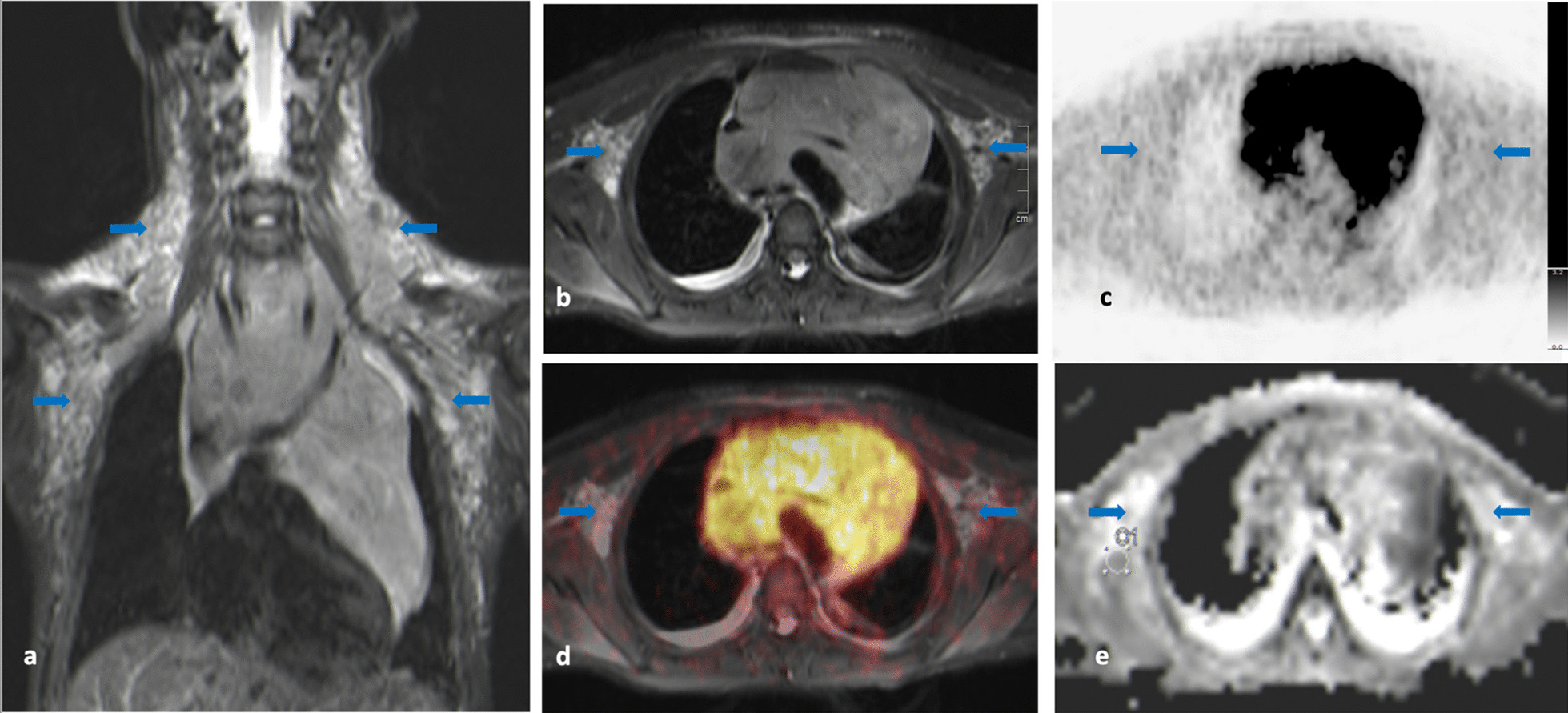
**a**–**e** Neck, axillae and chest on T2-TIRM image in coronal view (**a**); transversal view of axillae and chest on T2-TIRM (**b**), [^18^F]-FDG-PET (**c**), [^18^F]-FDG-PET/MR (**d**) and DWI at b-value of 800 (**e**): Lymphoma masses on both sides of the neck, with multiple, small lymph nodes extend caudally and filling both axillae (blue arrows) (**a**,** b**). In addition, diffuse lymphoma involvement of the thymus can be detected (**a**). Axillary lymph nodes exhibit physiological glucose uptake (SUV_max_ right: 1.17; SUV_max_ left: 1.25) (**c**,** d**) without restricted diffusion (ADC right: 1693 × 10^–6^ mm^2^/s; ADC left: 1746 × 10^–6^ mm^2^/s) (**e**)

This patient presented with enlarged cervical lymph nodes in levels II, III and IV on both sides of the neck (Fig. 2a). In addition, there were numerous small (< 1 cm) lymph nodes continuously extending from the cervical level II into both axillae (Fig. 2a). These small nodes were embedded in fatty tissue, which exhibited a pathologic signal on T2-TIRM sequences (Fig. 2a+b). However, the small bilateral axillary lymph nodes did not show increased glucose metabolism (SUV_max_ right side: 1.17; SUV_max_ left side: 1.25) (Fig. 2c+d). Furthermore, diffusion was not restricted (ADC right: 1693 × 10^–6^ mm^2^/s; ADC left: 1746 × 10^–6^ mm^2^/s) (Fig. 2e).

Taken all information together (lymph nodes < 1 cm, no increased glucose metabolism, no diffusion restriction) it was decided to consider both axillae not to be involved.

##### Lung hilum (Fig. 3a–c, Table 3***)

**Fig. 3:**
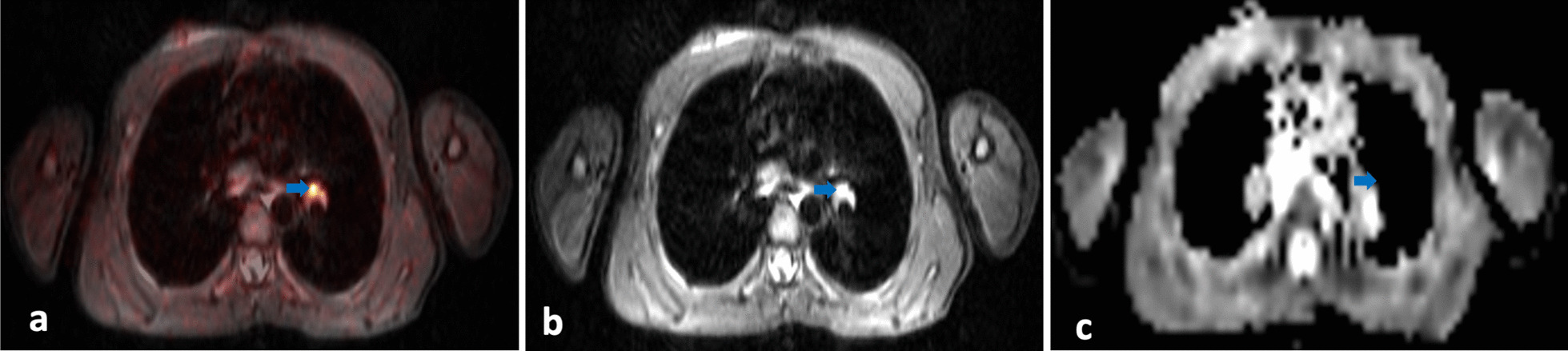
**a**–**c** [^18^F]-FDG-PET/MR image (**a**), T2-TIRM image (**b**) and DWI (**c**) of the thorax. The left hilar lymph node (blue arrow) has increased glucose metabolism (SUV_max_ 3.3) (**a**) but is small (0.9 × 0.7 cm) (**b**) and without diffusion restriction (ADC: 3800 × 10^–6^ mm^2^/s) (**c**)

Hilar lymph nodes are often affected, especially during respiratory tract inflammation and therefore may show moderately to markedly increased glucose metabolism as in this case (SUV_max_ 3.3) (Fig. 3a). However, neither patient history and clinical investigation nor whole-body [^18^F]-FDG-PET/MRI gave evidence of any active respiratory tract inflammation. Thus, an increased glucose metabolism of the left hilum lymph node was determined suspicious of lymphoma. However, on MRI the left hilar lymph node was small (0.9 × 0.7 cm) and diffusion was not restricted (ADC: 3800 × 10^–6^ mm^2^/s) (Fig. 3b+c).

This information combined (i.e., moderately increased glucose metabolism, but small lymph node and no diffusion restriction), the region was determined not involved.

#### Central nervous system (CNS)

None of the ten patients had CNS involvement according to cerebrospinal fluid (CSF) cytology. Correspondingly, [^18^F]-FDG-PET/MRI images were completely inconspicuous.

#### Waldeyer‘s ring (WR)

ENT exam performed by pediatric oncologists is a common way to decide on WR involvement [6].

WR involvement was suspected by ENT exam in two of our patients. However, the region appeared inconspicuous both on MRI and [^18^F]-FDG-PET (Table 1). On the other hand, there were two patients with negative ENT exam who both unequivocally were identified to have WR involvement on [^18^F]-FDG-PET and MR images (Table 1).

**Fig. 4:**
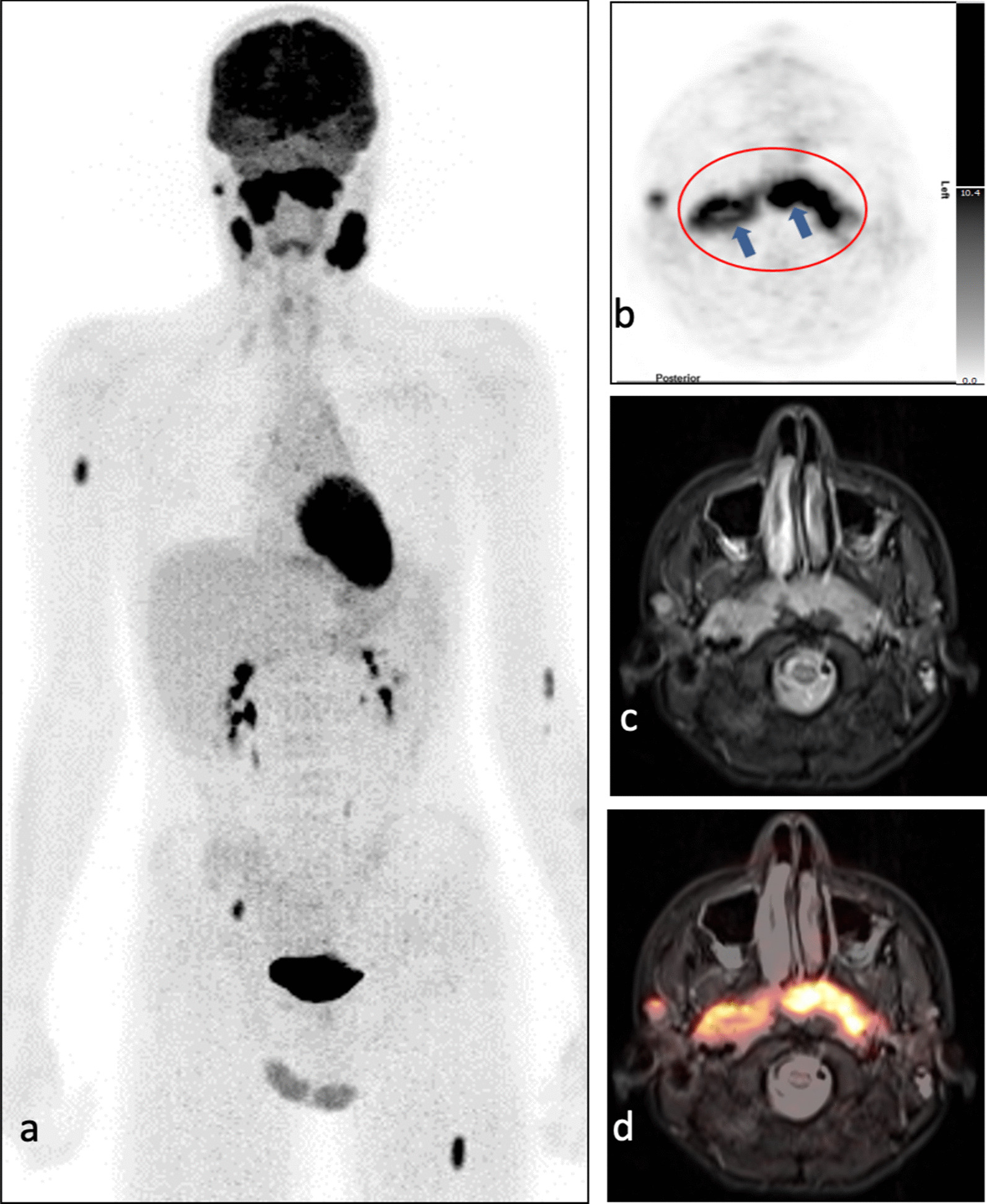
**a–d** The maximum intensity projection (MIP) shows extensive involvement of the Waldeyer's ring, bilateral cervical lymph node involvement, and three focal lesions in the skeleton (right humerus, right pelvis, left femur) (**a**). The pharyngeal tonsil (red rim) has asymmetric and inhomogeneous tracer uptake on [^18^F]-FDG-PET images (SUV_max_ right: 14.36; SUV_max_ left: 11.31) (blue arrows) (**b, d**). On T2-TIRM transversal MR images, the pharyngeal tonsil is markedly enlarged as a result of lymphoma involvement (**d**)

In Fig. 4 the images of one patient with negative ENT exam but diagnosis of WR involvement according to [^18^F]-FDG-PET/MRI are shown: The nasopharyngeal level (Fig. 4c+d) appears enlarged and the pharyngeal tonsil is configured irregularly (Fig. 4c+d). Glucose metabolism is markedly increased and distributed asymmetrically (SUV_max_ right: 14.36; SUV_max_ left: 11.31) (Fig. 4a+b).

#### Lungs

None of the ten patients had suspicion of lung involvement on [^18^F]-FDG-PET/MRI images, which was confirmed at least in two patients by additional chest CT.

#### Pleura

Four out of ten patients had pleural effusion without evidence of nodular pleural thickening on T2-TIRM images. However, on [^18^F]-FDG-PET images, two of them showed an area of increased glucose metabolism along the parietal pleura.

**Fig. 5:**
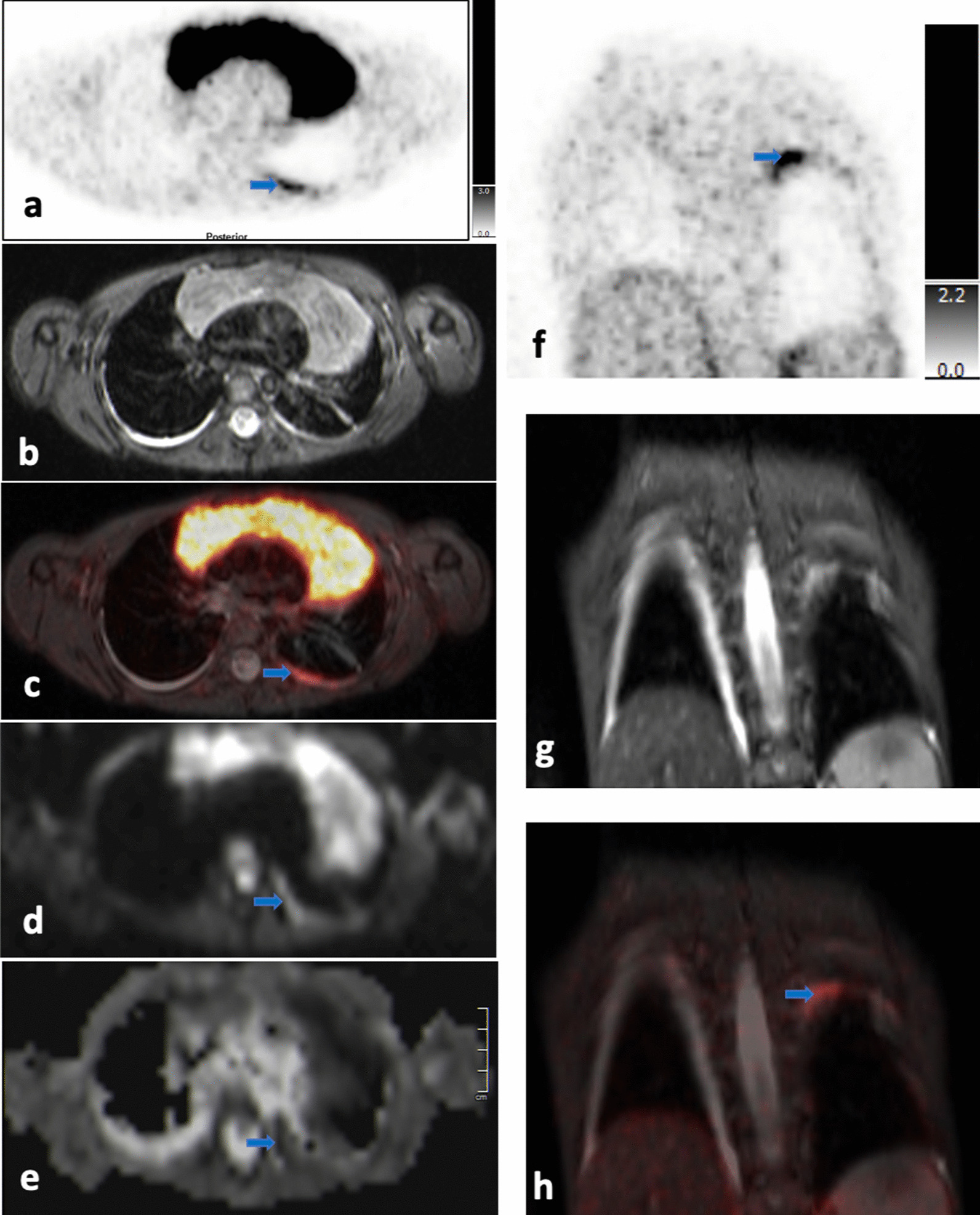
**a–h** [^18^F]-FDG-PET images transversal and coronal (**a, f**), MR T2-TIRM images transversal and coronal (**b,g**), [^18^F]-FDG-PET/MRI (**c,h**) and DWI at b-value of 800 (**d,e**) of the thorax. The blue arrows point to an area of the left posterior pleura with increased glucose uptake (SUV_max_ 2.88) (**a, c, f, h**) and a restriction in diffusion (ADC levels of about 636 × 10^–6^ mm^2^/s) (**d, e**). This area corresponds to pleura involvement which is not distinguishable from pleural effusion on MR T2-TIRM images (**b, g**)

[^18^F]-FDG-PET/MRI images from one of those two patients are displayed in Fig. 5: Corresponding to increased glucose metabolism in an area of the left posterior pleura (SUV_max_ left: 2.88; SUV_max_ right: 0.86) (Fig. 5a, c, f, h), diffusion was restricted in this area as well (ADC levels of about 636 × 10^–6^ mm^2^/s) (Fig. 5d+e). In keeping with this, malignant cells (96%) were detected during thoracentesis (Table 1). However, distinction between pleural effusion and lymphoma manifestation of the pleura remained impossible on MRI T2-TIRM images, also on re-evaluation (Fig. 5b+g).

#### Spleen

In concordance with ultrasound, splenic parenchyma of all ten patients was inconspicuous both morphologically (i.e., no focal lesions detectable on MRI) and metabolically (i.e., tracer uptake of the spleen < tracer uptake of the liver and/or the BM). However, in one patient ultrasound and MRI concluded splenomegaly.

#### Liver

None of the ten patients had suspicion of liver involvement on [^18^F]-FDG-PET/MRI which was in accordance with ultrasound.

#### Kidneys

Ultrasound as well as MRI and [^18^F]-FDG-PET concordantly identified two patients with bilateral lymphoma manifestations of the kidneys.

**Fig. 6:**
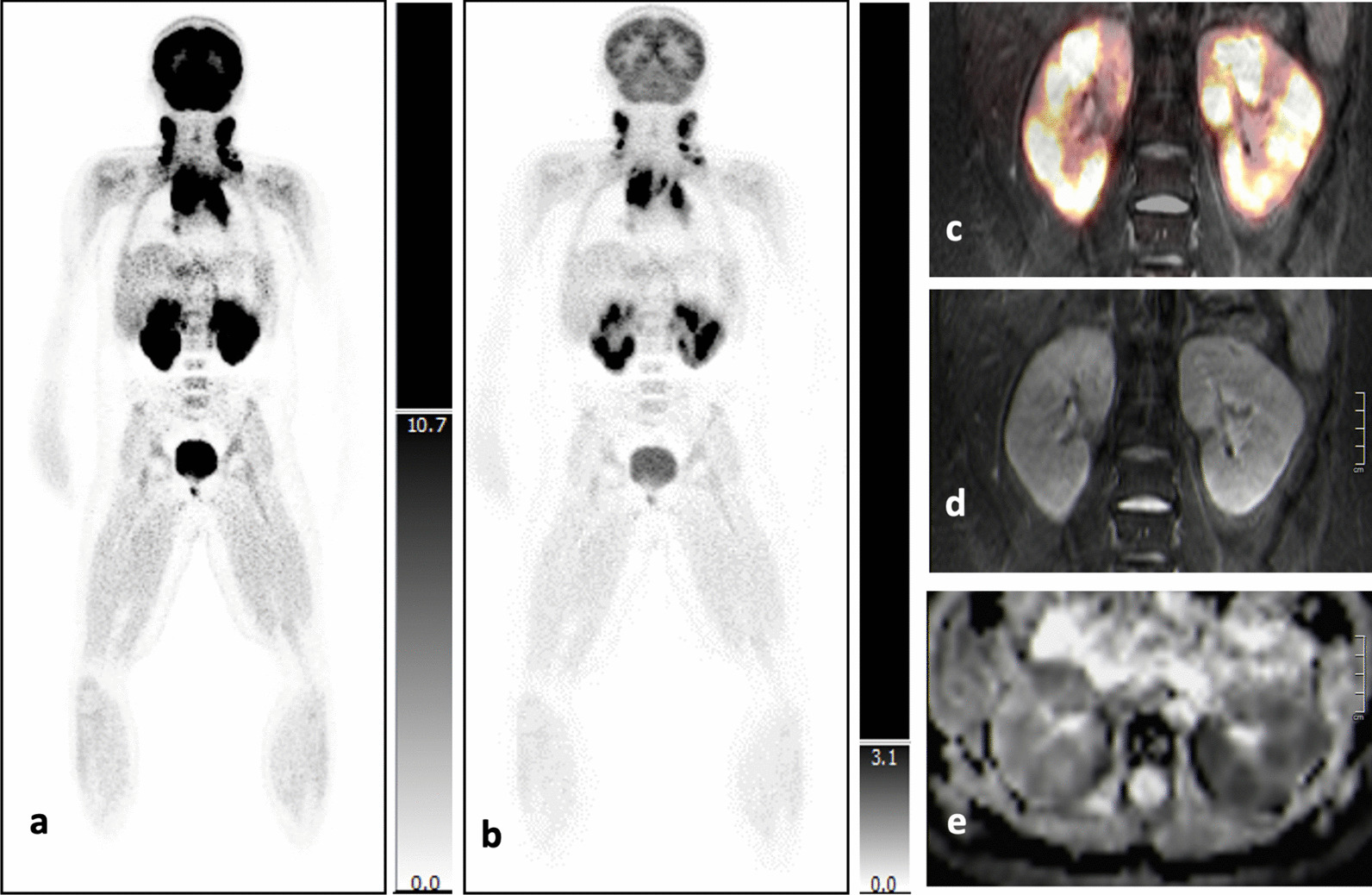
**a–e** Coronal [^18^F]-FDG-PET slices with different intensity levels (**a, b**) show the relation between radiotracer accumulation in the bladder (SUVmax 12,8) and both involved kidneys (SUVmax right: 38,02; SUVmax left: 42,91). Coronal fusion images display multiple, metabolically active lesions in both kidneys (**c**). On coronal MR T2-TIRM images both kidneys are enlarged and corticomedullary junctions are blurred (**d**). Multiple foci in both kidneys are almost restricted in diffusion (ADC about 908 × 10–6 mm2/s) (**e**)

Figure 6 shows imaging from one of the two patients: [^18^F]-FDG-PET reveals multiple areas of markedly increased glucose uptake (SUV_max_ right kidney: 38.02; SUV_max_ left kidney: 42.91; SUV_max_ bladder: 12.8 for comparison) in both organs (Fig. 6a–c). On T2-TIRM images, the involved kidneys appear enlarged with blurring of the corticomedullary junctions (Fig. 6d). ADC values on DWI reached nearly pathologic values at about 908 × 10^–6^ mm^2^/s (Fig. 6e).

#### Skeleton

Six of the ten patients were identified to have skeletal involvement (one DLBCL, two primary osseous NHL, one Burkitt lymphoma, two T-cell lymphoma). In five patients, only [^18^F]-FDG-PET/MRI was indicative of involvement. In one patient, both [^18^F]-FDG-PET/MRI and bone marrow (BM) biopsy showed involvement.

**Fig. 7:**
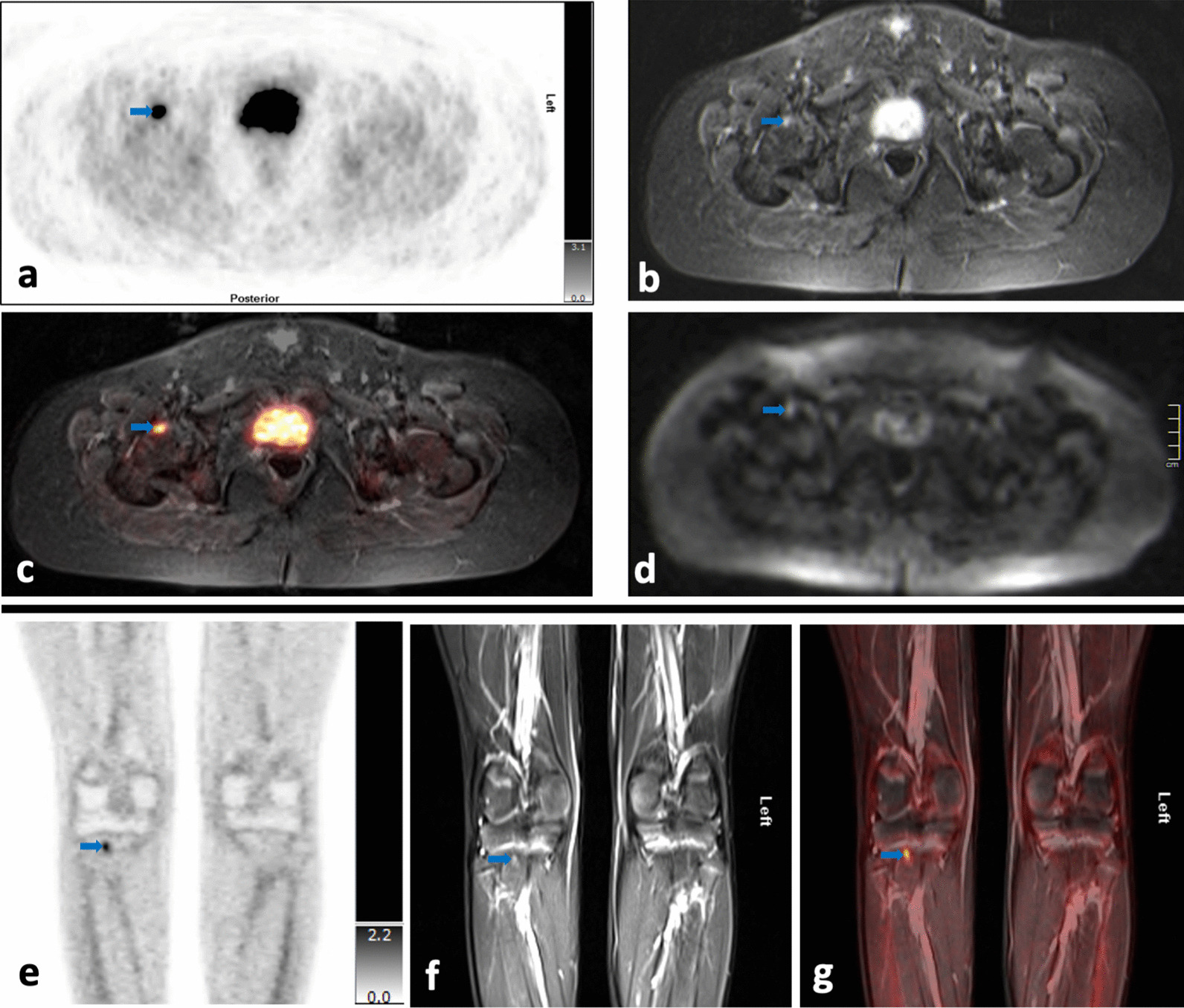
**a–g** Upper double row: Small focus of skeletal involvement in the right femoral head which has increased glucose metabolism on [^18^F]-FDG-PET images (SUV_max_ 5.98) (**a, c**), is hyperintense on MR T2-TIRM images (**b**) and shows restricted diffusion on DWI (**d**). Lower row: Small focus of skeletal involvement in the right proximal tibia which has moderately increased glucose metabolism on [^18^F]-FDG-PET images (SUV_max_ 2.18) (**e, g**) and is slightly hyperintense on MR T2-TIRM images (**f**)

In the six patients, a total of 13 skeletal lesions were detected on [^18^F]-FDG-PET/MRI. These comprised six lesions accompanied by local bone destruction, and seven lesions confined to BM exclusively. All six lesions with local bone destruction were detected on [^18^F]-FDG-PET and MRI during the first run. Two of the seven lesions confined to BM were first missed on MRI analysis. However, after knowing the localization of increased glucose metabolism, correlating very small lesions could be identified on MRI (on TIRM sequences and on DWI) (Fig. 7).

**Fig. 8:**
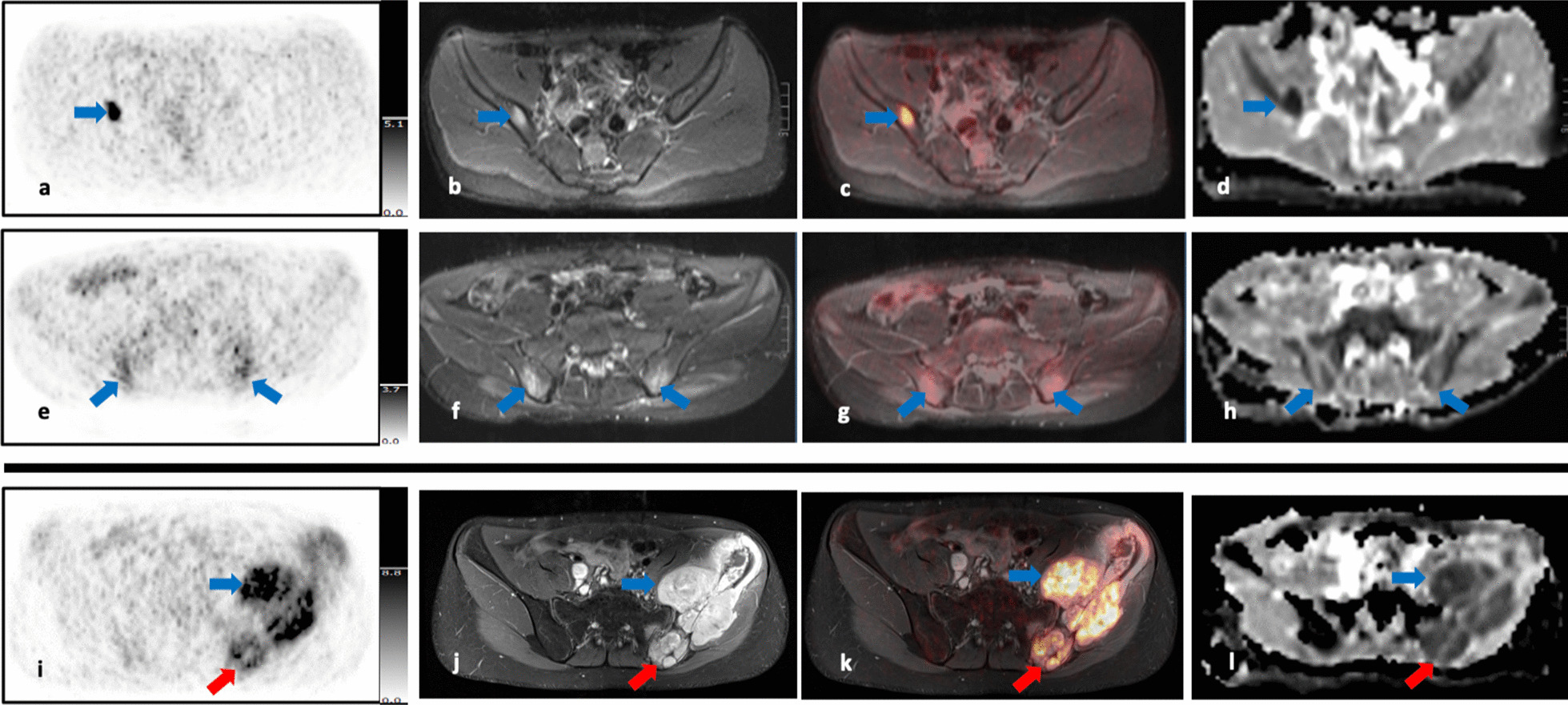
**a–l** Images from the upper and the middle row belong to the same patient, whereas images from the lower row belong to another patient. The upper row (**a–d**) shows images of a single lesion in the middle of the right iliac bone (blue arrow). The lesion has increased glucose metabolism on [^18^F]-FDG-PET images (SUV_max_ 9.80) (**a, c**), is hyperintense on MR T2-TIRM images (**b**) and has restricted diffusion on DWI (ADC about 419 × 10^–6^ mm^2^/s) (**d**). The middle row (**e–f**) shows images including the biopsy sites on both posterior iliac crests (blue arrows): Both biopsy sites have moderate and diffuse radiotracer uptake on [^18^F]-FDG-PET images (SUV_max_ right: 2.65; SUV_max_ left: 2.74) (**e,g**), are moderately hyperintense on MR T2-TIRM images (**f**) and do not show restriction in diffusion on DWI (ADC right: about 1240 × 10^–6^ mm^2^/s; ADC left: about 1148 × 10^–6^ mm^2^/s) (**h**). The third row shows extensive lymphoma involvement of the left iliac bone and the biopsy channel (**i-l**): Lymphoma manifestation from the left iliac bone have markedly increased glucose metabolism on [^18^F]-FDG-PET images (blue arrows) (**i, k**), are hyperintense on MR T2-TIRM images (**j**) and markedly restricted in diffusion on DWI (**l**). The biopsy channel (red arrows) can be distinguished from the lymphoma lesions by lack of radiotracer uptake (**i, k**). It is hypointense on MR T2-TIRM images (**j**) and shows no diffusion restriction on DWI (**l**)

The patient in whom BM biopsy was positive had extended lymphoma involvement of the left pelvic bones and the needle track ran directly through the lymphoma manifestation (Fig. 8i–l).

Pertaining to the five patients whose BM biopsy was negative despite of skeletal lymphoma involvement it is noteworthy that one focal skeletal lesion was not far (approximately 4.2 cm) located from the biopsy site (Fig. 8a–h).

## Discussion

A new staging system for pediatric NHL was released in 2015 [2]. The respective publication also mentions the developments of cross-sectional imaging over the past decades including the latest which is [^18^F]-FDG-PET/MRI [2]. We performed a single-center evaluation with ten pediatric NHL patients at the time of initial diagnosis in order to investigate the performance of [^18^F]-FDG-PET/MRI by using a limited number of MRI sequences. A focus was put on lymph node regions as well as organs and several extranodal sites of potential lymphoma involvement.

To date publications evaluating the application of [^18^F]-FDG-PET and MRI (including DWI) in NHL patients stem from imaging procedures performed separately of each other. Thereby, time intervals of up to two weeks are described [7–9]. However, during such time intervals lymphoma may have further developed and spread. Furthermore, other non-malignant diseases like tonsillitis or pneumonia may occur or resolve in the meantime and could lead to differing results. In this respect, a comparison is not valid. In contrast to that, simultaneous [^18^F]-FDG-PET/MRI allows imaging without any delay between the two modalities and therefore a more reliable comparison of the respective findings.

In our evaluation, a total of 190 lymph node regions were assessed. For 186 of 190 (98%), [^18^F]-FDG-PET and MRI came to concordant results. This is in line with the literature describing high [^18^F]-FDG-PET avidity for most lymphoma subtypes and excellent lymph node accessibility with whole-body MRI [10–12]. For the few cases with discrepant findings the addition of diffusion weighted images (DWI) facilitated decision making. Using the cutoff value at 800 × 10^–6^ mm^2^/s for non-Hodgkin lymphoma, which was also suggested by Kwee et al. [13] and Vermoolen et al. [14], resulted in a clear differentiation between likely and unlikely lymphoma involvement. We were aware that motion artifacts, which often occur in the areas of neck, chest and abdomen, may impair the reliability of DWI and ADC values [15]. In our limited cohort, however, such problems were not noticed which may be due to the increased efforts in handling pediatric patients (e.g., sufficient immobilization during [^18^F]-FDG-PET/MRI acquisition or the use of respiratory triggering) [3]. Thus, further evaluation of the potential capacity of DWI seems warranted in order to establish this functional information as a third cornerstone beside morphology and metabolism.

The Waldeyer’s ring (WR) turned out to be a region with noticeable discrepancies between clinical examination and [^18^F]-FDG-PET/MRI: In two patients, suspicion of WR involvement based on ENT exam could not be confirmed on [^18^F]-FDG-PET/MR images. On the other hand, there were two patients showing highly suspicious pattern of their WR on [^18^F]-FDG-PET/MRI with unremarkable ENT exam. Those discrepancies can result from different facts: First, ENT exam as an inspection of the oral cavity, regularly performed by pediatric oncologists, can only evaluate the oro-pharynx. The nasopharynx (e.g., pharyngeal tonsil) remains unevaluated if not examined by an ENT specialist using dedicated instruments. Second, even if the entire WR is examined by an ENT specialist, only the mucosal surface is evaluated but not the underlying tissue. Furthermore, alterations of the mucosa can be unspecific and therefore difficult to interpret. This makes ENT examinations highly subjective and therefore prone to interobserver variability [6, 16]. Thus, an evaluation of the WR should always include cross-sectional imaging, preferably [^18^F]-FDG-PET/MRI. The detection of WR involvement is crucial to anticipate airway obstruction early on especially in patients with NHL entities often affecting the WR such as Burkitt lymphoma and DLBCL [16, 17]. Furthermore, there is evidence that NHL patients with WR involvement may benefit from irradiation of their WR [18, 19].

Concerning pleura involvement, the results of our study support previous assumptions that [^18^F]-FDG-PET can differentiate between pleural effusion and malignant pleura involvement [20]. Pleural assessment, however, is challenging on whole-body MR sequences, especially if solely coronal slices are acquired [21, 22]: Due to the thoracic convexity, coronal slices are prone to partial volume effects. Partial volume effects considerable complicate the assessment of the pleura, so that small lesions in particular are easily missed. On transversal slices, by contrast, partial volume effects are less frequent, making them more suitable for pleural assessment. Another option would be a dedicated lung MRI. However, this would significantly prolong imaging time and contradicts the intention of whole-body MRI which is to provide a fast overview without loss of compliance in young patients due to long imaging time.

Splenic involvement occurs in about 30–40% of all NHL patients [23]. However, in the cohort analyzed here, none of the patients had suspicious focal lesions within splenic parenchyma, neither on ultrasound nor on MRI or [^18^F]-FDG-PET. Nevertheless, in one patient splenomegaly was detectable. Splenomegaly is challenging because it is a relatively unspecific sign. It can accompany spleen involvement as well as various pathologies like infectious diseases (e.g., mononucleosis, malaria), metabolic disorders (e.g., Morbus Gaucher) or liver diseases resulting in portal hypertension [24]. In case of normal parenchymal texture some publications suggest comparing radiotracer uptake of the spleen with liver and/or bone marrow uptake. If tracer uptake of the spleen exceeds tracer uptake of liver and/or bone marrow, it is recommended to assume spleen involvement [25, 26]. In our patient with splenomegaly on ultrasound, radiotracer uptake of the spleen did not exceed that of the liver or the bone marrow.

Kidney involvement was detected in two of the ten patients. Both patients had bilateral renomegaly due to extensive lymphoma infiltration. In young NHL patients with kidney involvement, multiple bilateral large lesions are the most commonly found pattern [27, 28]. Both MRI (including DWI) and [^18^F]-FDG-PET were able to correctly detect kidney involvement. This is important to mention for [^18^F]-FDG-PET, since kidney evaluation with [^18^F]-FDG-PET is often regarded as extremely challenging due to renal excretion of the radiotracer. However, cortical lesions and manifestations distant from the collecting system are well distinguishable from physiological tracer excretion as it was in our two patients.

Skeletal involvement in NHL patients can be either focal or diffuse. Literature review suggests a pattern of rather focal involvement in patients with DLBCL, whereas other NHL entities tend toward diffuse bone marrow infiltration [29–31].

Bone marrow biopsy is much more sensitive in detecting diffuse BM infiltration compared to [^18^F]-FDG-PET and MRI [32–35]. In contrast, a focal involvement pattern is detectable on [18F]-FDG-PET and MR images with the highest sensitivity [32–34]. All six patients from our cohort who had skeletal involvement (only one of them had DLBCL) exhibited a pattern of focal involvement which was well detectable through [^18^F]-FDG-PET/MRI. However, BM biopsy taken from the iliac crest was negative in five out of the six cases. It is worth mentioning that BM biopsy was also negative in the patient in whom one of the BM lesions was not far located from the needle track. This suggests that BM biopsy probably only yields a positive result if the biopsy needle hits the lesion visible on imaging, which, in turn, may explain the low sensitivity of bone marrow biopsies in case of a focal involvement pattern.

 In one of the six patients [^18^F]-FDG-PET/MRI and BM biopsy were positive. This constellation could be of prognostic value: Based on an analysis of 327 adult DLBCL patients, Cerci et al. [36] found that patients who were positive in both modalities, [^18^F]-FDG-PET and BM biopsy, had a worse outcome compared to patients who were positive in only one modality. However, in our patient the needle track ran directly through the lymphoma manifestation. Thus, in this patient positivity of both modalities does not necessarily imply a worse outcome.

Several limitations of this retrospective evaluation need to be mentioned:

First, the number of cases is small. Thus, the described results should be confirmed, preferably within a large multicenter trial with predefined image acquisition parameters.

Second, the investigated cohort does not cover the typical spectrum of childhood NHL entities, which are Burkitt lymphoma, lymphoblastic lymphoma and anaplastic large-cell lymphoma. The spectrum of this study refers to an adolescent population in which DLBCL is more common.

Third, our study population does not seem to include high-risk patients. Particularly LDH, as one of the main risk factors, was relatively low in all cases.

Forth, some of the potential sites of extranodal NHL involvement like CNS, liver and lung were not evident in the analyzed cohort, precluding the assessment of the full potential of [^18^F]-FDG-PET/MR imaging.

Fifth, despite being able to show that the applied DWI performed excellent, it has to be mentioned that new developments concerning DWI have evolved, e.g., whole-body-DWI with background suppression (DWIBS) [37]. The latter provides superior local resolution and reduced artifact susceptibility [38]. It is characterized by a relatively short acquisition time, which is particularly desirable in the setting of whole-body imaging in children and adolescents [39]. And a robust fat suppression has a positive effect on subsequent three-dimensional reconstruction. In young children, however, the physiological diffusion restriction of the pelvis and spine has to be taken into account, since it could lead to false positive findings [39]. New opportunities to display DWI findings (e.g., MIP grey-scale inverted DWI) might also facilitate direct comparison between function and metabolism.

Sixth, the clinical and paraclinical results were used as standard of reference to some of the imaging results. However, clinical and paraclinical parameters have limitations as well. To overcome these limitations, confirmation through biopsy would be necessary. The latter, however, is often not feasible nor justifiable in the clinical setting as well as ethically.

Seventh, the aim of this study was to evaluate the performance of whole-body [^18^F]-FDG-PET/MRI for a limited overall acquisition time. It was not the aim to compare [^18^F]-FDG-PET and MRI. For that, a broader spectrum of MRI sequences would have been necessary leading to a considerable prolongation of image acquisition time, which is difficult in a pediatric age group. However, imaging technology is developing quickly. Thus, in the future a broader spectrum of MR sequences can be expected to be acquired within one hour of acquisition time.

## Conclusion

Despite the small cohort investigated here, we were able to show that whole-body [^18^F]-FDG-PET/MR is a valuable staging tool in pediatric NHL, especially when it comes to the evaluation of the skeleton, the Waldeyer’s ring, pleura and lymph nodes. Thereby, morphologic, metabolic and functional (DWI) information complement each other well.

## Data Availability

The datasets generated and analyzed during the current study (SPSS tables) are available from the corresponding authors upon request.

## Abbreviations

ADC: Apparent diffusion coefficient
BM: Bone marrow
CNS: Central nervous system
CT: Computed tomography
DLBCL: Diffuse large B-cell lymphoma
DWI: Diffusion weighted images
DWIBS: DWI with background suppression
EANM: European association of nuclear medicine and molecular imaging
ENT: Ear, nose, throat
[^18^F]-FDG-PET/CT: 2-Deoxy-2-[^18^F]fluoro-d-glucose positron emission tomography/computed tomography
[^18^F]-FDG-PET/MRI: 2-Deoxy-2-[^18^F]fluoro-d-glucose positron emission tomography/magnetic resonance imaging
HL: Hodgkin lymphoma
IPNHLSS: International pediatric non-Hodgkin lymphoma staging system
LDH: Lactate dehydrogenase
NHL: Non-Hodgkin lymphoma
TIRM: Turbo-inversion recovery magnitude
WR: Waldeyer’s ring
